# BOLD contrast: A challenge for cardiac image analysis

**DOI:** 10.1186/1532-429X-18-S1-W27

**Published:** 2016-01-27

**Authors:** Ilkay Oksuz, Marco Bevilacqua, Anirban Mukhopadhyay, Rohan Dharmakumar, Sotirios Tsaftaris

**Affiliations:** 1 grid.4305.20000000419367988The University of Edinburgh, Edinburgh, United Kingdom; 2 IMT Lucca, Lucca, Italy; 3 grid.50956.3f0000000121529905Cedars-Sinai Medical Center, Los Angeles, CA USA; 4 grid.412041.2000000012106639XUniversity of Bordeaux, Bordeaux, France

## Background

Blood oxygen level dependent (BOLD) imaging of the heart has been shown to assess ischemia at rest or benign non-invasive stress. However, since BOLD changes are not readily visible to the naked eye, post processing and analysis is necessary. But, BOLD contrast is modulated by the presence of disease and also by the imaging protocol and sequence, posing significant challenges in creating robust algorithms for analyzing such data.

We discuss recent approaches for automatically segmenting and registering cardiac-phase resolved CP-BOLD MRI studies.

## Methods

**Figure 1:**
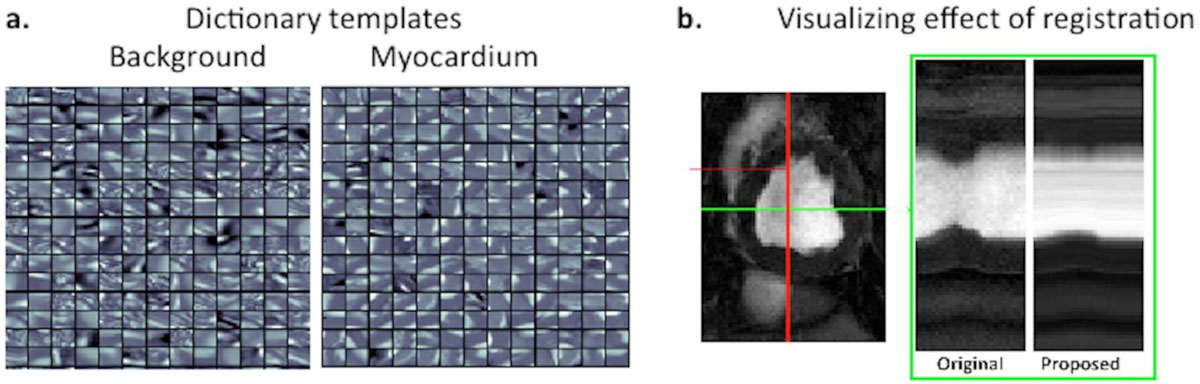
**(a) A set of dictionary templates for Background and Myocardium learnt from patches of size 11 by 11**. Observe how each template appears to describe the structure that it represents. For example in the myocardium class, one can see parts of the myocardium under different poses. **(b)** For the visualization on the right we project intensity values on the vertical line shown on the left. Without registration we clearly see the motion of the myocardium; however, using the proposed algorithm this motion is largely compensated for.

We use CP-BOLD data from controlled canine (10) experiments imaged at rest, under baseline and severe LAD occlusion (ischemia). We delineate manually all myocardial borders to have ground truth. Based on these delineations we learn dictionaries (examples shown in Figure 1a) to describe a given patch (a small square image block) with as few (ie., sparse) linear combinations of templates as possible. The coefficients of those combinations form a feature vector for each patch, and can describe it while remaining unaffected by the BOLD contrast. We use such feature vectors for myocardial segmentation by optimizing a non-linear classifier but also for non-linear registration to correct for myocardial motion in this cine-type acquisition. We define, and use within our algorithm, a new similarity metric that compares how close those features are between patches belonging to target and reference images.

### Evaluation

We follow a leave 1 out crossvalidation strategy: we use 9 canines to learn dictionaries and we use our methods to segment and register the images of the other canine. (We do this separately for baseline and ischemia). We use the Dice metric that measures overlap between the automatically obtained segmentation vs. the ground truth. We use Dice to also evaluate registration by propagating the segmentation mask of end diastole (reference) of the cardiac cycle to all others (target) and compare the propagated mask with the one in the ground truth.

## Results

Our segmentation approach achieves on average (std) a dice score of 75(2) at baseline and 71(2) during ischemia. Our registration, on average achieves a Dice score of 66(9) for baseline and 60(13) for ischemia. Compared to methods from the literature we are 20%, and 5% better in segmentation and registration accuracy respectively. Figure 1b, shows that registration can compensate for myocardial motion.

**Figure 2:**
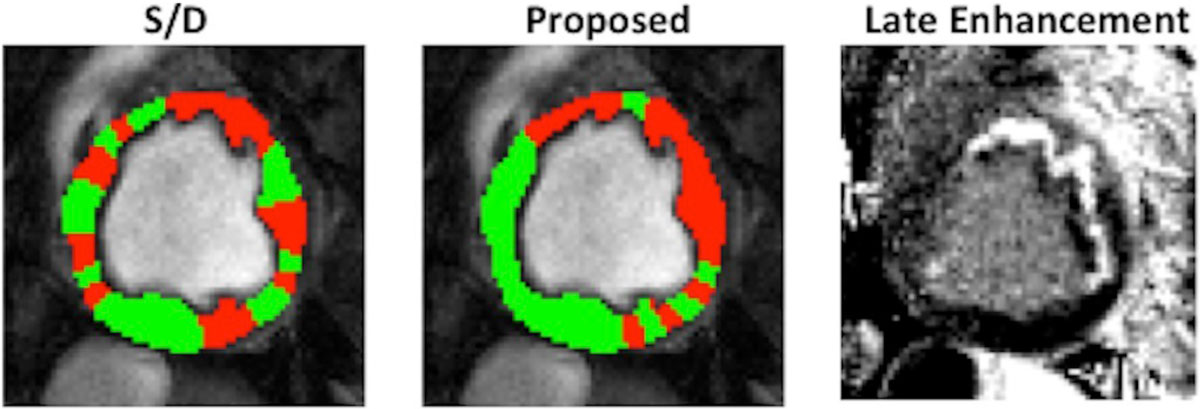
**On the left we show the result of estimating presence of ischemia at rest based on the S/D approach of Tsaftaris et al., Circ Imaging, 2013, which relies on the ratio of intensity at systole over diastole**. In the middle we show the outcome when using the algorithms presented here to register the myocardium and find correspondences across the cardiac cycle and use those correspondences within the framework developed by Bevilacqua et al, IEEE Trans. Med. Imaging, 2015. On the right we show for the same canine a late enhancement image obtained after 3 hours of occlusion and during reperfusion, confirming the presence of an infarcted territory close to the LAD. Our algorithm captures well the corresponding area at risk, much better than S/D.

Figure 2 illustrates how we can better detect ischemia by incorporating via registration information across the cardiac cycle.

## Conclusions

We outlined a series of algorithms that can segment, register and analyze these datasets. While more evaluation is necessary, BOLD does provide unique opportunities to study the heart without any contrast and stress.
